# International Air Travel to Ohio, USA, and the Impact on Malaria, Influenza, and Hepatitis A

**DOI:** 10.1155/2016/8258946

**Published:** 2016-03-31

**Authors:** Donald E. Brannen, Ali Alhammad, Melissa Branum, Amy Schmitt

**Affiliations:** ^1^Greene County Public Health, 360 Wilson Drive, Xenia, OH 45385, USA; ^2^Wright State University, Dayton, OH 45435, USA; ^3^Xavier University, Cincinnati, OH 45207, USA; ^4^Division of Aerospace Medicine, Boonshoft College of Medicine, Wright State University, Dayton, OH 45435, USA; ^5^Royal Saudi Arabian Armed Forces Medical Services, Jeddah 21577, Saudi Arabia

## Abstract

The State of Ohio led the United States in measles in 2014, ostensibly related to international air travel (IAT), and ranked lower than 43 other states in infectious disease outbreak preparedness. We conducted a retrospective cohort study using surveillance data of the total Ohio population of 11 million from 2010 through 2014 with a nested case control of air travelers to determine the risk of malaria, seasonal influenza hospitalizations (IH), and hepatitis A (HA) disease related to international travel and to estimate the association with domestic enplanement. IAT appeared protective for HA and IH with a risk of 0.031 (.02–.04) but for malaria was 2.7 (2.07–3.62). Enplanement increased the risk for nonendemic M 3.5 (2.5–4.9) and for HA and IH 1.39 (1.34–1.44). IAT's ratio of relative risk (RRR) of malaria to HA and IH was 87.1 (55.8–136) greater than 219 times versus domestic enplanement which was protective for malaria at 0.397 (0.282–0.559). Malaria is correlated with IAT with cases increasing by 6.9 for every 10,000 passports issued.

## 1. Introduction

Ohio led the United States in measles cases in 2014, ostensibly and directly related to unvaccinated international air travelers (Ohio Department of Health, 2015). Researchers have validated concerns that Ohio scored lower than 43 other states in being prepared for infectious disease outbreaks (Trust in America's Health, Robert Wood Johnson Foundation, news release, Dec. 17, 2015). These researchers have urged efforts to protect Americans from “new threats such as Middle East respiratory syndrome coronavirus (MERS-CoV) and antibiotic-resistant superbugs, along with resurging diseases such as tuberculosis, whooping cough and gonorrhea.” These concerns over emergent diseases led us to review the literature of infectious diseases with adequate travel histories and to conduct a retrospective cohort study of the risk to Ohio's population including a nested case control study to explore the association between infectious disease risk and air travel. The methodological reasons for selecting malaria, seasonal influenza hospitalizations (IH), and hepatitis A (HA) among the diseases reviewed will be discussed. HA virus is highly contagious and can affect liver function. HA is transmitted frequently through contaminated food and water. The World Health Organization (WHO) estimates that there are over 1.3 million cases of acute HA every year. Influenza disease is caused by a virus that attacks the respiratory system. Symptoms include sudden onset of fever, body aches, fatigue, and cough. Pneumonia can be a complication of influenza, especially in the persons with weakened immune systems. WHO estimates that over 3 million persons are hospitalized and over 250,000 persons die each year from influenza.

## 2. Method

### 2.1. Literature Review of Infectious Diseases with Adequate Travel History

Disease transmission during air travel has been a concern for many years. Historically, the spread of nonendemic infectious disease has been linked to air travel contingent on the weather [1]. Specifically, the risk of viremic transmission has been directly related to the magnitude of* viremic person-days* the arriving traveler spends in the country during the season the “capable-vector” is active. For example, the months the* Aedes* mosquito is active and able to bite viremic air travelers arriving from endemic areas would contribute to disease spread. The* Aedes* mosquito is found throughout the world except Antarctica. Several species are especially important to note including* Aedes aegypti* and* Aedes albopictus*. The* Aedes aegypti* transmit several flaviviruses including dengue, yellow fever, chikungunya, and Zika. This species cohabitates with humans so it is difficult to eradicate. The* Aedes aegypti* is a tropical mosquito but the range is slowing expanding to more moderate climes. The* Aedes albopictus*, known as the Asian Tiger mosquito, has adapted from a northern forest environment to live in suburban areas. After a blood meal females lay eggs in water. Mosquitoes transmit disease mainly by biting an infected person and having their salivary glands infected with the virus, bite a second uninfected person.* Aedes albopictus *can transmit chikungunya and dengue. The Ohio Department of Health reports that the* Aedes aegypti* is not established in Ohio but the Asian Tiger mosquito was introduced in the United States in 1985 and has spread through much of the country. With expansion of air travel networks the risk of importing diseases increases from both people and vectors readily connected to endemic malaria regions of the world [2, 3]. However, the actual public health burden of the impact of air travel on infectious disease is not known.

Tracking of cases can indicate the origin of the outbreak [4]. The decision for tracing back cases can be done if there are positive cases during flight or if the symptoms develop within the incubation period after travel [5]. If an asymptomatic air traveler commutes wide distances, there is by no means a certainty that once the case is recognized these containment measures would be appropriately taken. For instance, a case study following an international flight after exposure to measles had variable effectiveness using contact tracing to identify those exposed. Illustratively, Ohio led the United States in measles cases in 2014, directly related to unvaccinated international air travelers (Ohio Department of Health, 2015). In the same year a healthcare worker exposed to Ebola traveled to Ohio [6].

There are challenges to public health agencies associated with control of infectious disease following a transatlantic flight after disease transmission including late diagnosis, tracking of passengers, and cooperation among various international and domestic agencies [7]. If control measures are not instituted at the start time of a pandemic, transmission will quicken. In an emergent viral illness, genetic reassortment and health status impact infectivity [8]. The role of travel and healthcare resources predicted the spread of pandemic influenza [9]. In the initial spreading phase containment was possible with enhanced surveillance and rapid deployment of control measures. Improved management through organizational culture of public health agencies during the H1N1 pandemic could have prevented 7,500 virus hospitalizations in the United States saving 45 million dollars.

The effect of international air travelers, air crews, and mode of conveyance as a source and continued spread of latent infectious disease during and after flight is a major concern for global health [10, 11]. The aerospace vehicle cabin presents a risk for passengers and crew as microbes are concentrated in the closed environment [12–14]. Specifically short and latent diseases involved such as cholera with an incubation of 2 hours to 5 days and highly infectious drug resistant tuberculosis with decade long latencies have infected passengers [4, 15]. Even with screening inflight transmission can occur due to varying incubation periods [16].

The effect of international and frequent travel on global contagion and epidemic source has been modeled using a spreading pattern in presence of random fluctuations [17, 18]. A probabilistic model of severe acute respiratory disease has been used to forecast the worldwide spread of infectious diseases and epidemics [19]. Models of simplified air transport routes have been adequate to assess infectious disease transmission [20]. Even screening of arriving passengers has not been as effective rather it has been the control measures in place and on the intensity of air travel between regions that impacted the number of severe acute respiratory syndrome (SARS) cases [21]. Once a highly infectious human-to-human transmissible agent reaches the top 50 global airports, the spread around the world is unstoppable [22]. The use of immediate travel restrictions including air, land, and sea, up to 99%, was estimated to delay the epidemic peak to allow for a vaccination campaign and other pandemic control measures to be instituted [23].

The role of airports and airlines in the transmission and spread of vector-borne diseases was helpful in predicting the risks of vector-borne disease importation and establishment [24, 25]. This model is consistent with cases of “airport malaria” which found the greatest risks of a* Plasmodium falciparum*-carrying mosquito being imported through air travel [26]. Using international ship and aircraft traffic combined with climatic information, the historical spread of* Aedes albopictus* was used to show disease vector spread along the global transportation network [27]. Disease vectors now have the ability to become established across great distances in short periods of time due to global air travel. More research is needed due to other factors like the overuse of antibiotics, intensive agriculture, climate change, high population densities, and inadequate water treatment facilities [28].

We initially hypothesized that international air travel of infected travelers increases the risk of infectious disease in nonendemic areas. After an exhaustive literature review of air travel and infectious disease we started a practical review of what diseases of public health concern had either high virulence or incubation times that had adequate travel histories. Initially we did not automatically rule out any of the communicable diseases on the reportable list. On April 1, 2015, we developed a taxonomy to identify the diseases by symptoms, lab tests, and epidemiological links to other cases. This led us to select the diseases we felt would be of most concern. In an ascending order of incubation periods the candidate diseases were influenza, SARS, MERS, Ebola, dengue, Marburg, malaria, and mumps. Mumps and measles, both vaccine preventable diseases, dropped out as candidates due to their low occurrence among the 88 counties in Ohio, with only sporadic but important increases, would be difficult to statistically study. Ebola, SARS, and Marburg were so rare; they would also be difficult to study. Malaria, IH, and HA did have some regular occurrence and had another benefit: they each had different autochthonous presentations in the Ohio cohort, with IH being seasonal, HA mostly from endemic rather than imported by persons (although imported products could cause the disease), and malaria being almost entirely from an area where international air travel is required.

Explanatory factors will be also examined including whether there is an increased trend over time of vector versus nonvector related diseases; whether endemic diseases are greater than nonendemic disease in regard to local air travel boarding; and whether there is a correlation of the rate of malaria with international air travel. While our initial purpose was to explore the risk of infectious diseases to air travel we first had to ask what diseases should we select to study. As noted above our taxonomy key to identify agents of concern led us to consider incubation and transmission factors. However, not only did the disease have to be of global concern there had to be an incidence of the disease in the geographic location of interest. That is, the disease had to have a sufficient number of cases to be of concern in Ohio. Also the travel histories would have had to be collected prospectively in order to be studied. This left the three diseases of interest: IH, HA, and malaria. The overall objective of the study is to determine the risk of malaria, seasonal IH, and HA disease related to international travel and to estimate the association with domestic enplanement.

### 2.2. Study Area, Cohort, and Ethical Approval

This project was submitted to Wright State University Institutional Review Board and was exempted from review. The Ohio Disease Reporting System (ODRS) is part of the United States National Electronic Disease Surveillance System (https://wwwn.cdc.gov/nndss/nedss.html). ODRS is a population based disease surveillance system which facilitates electronic transfers of public health surveillance data from the healthcare system to public health departments. Using a mix of cohort and case control methods on the entire cohort of Ohio, ODRS exposure variables and census data were analyzed. The cohort was the entire eleven million population of Ohio with controls being those persons who are not sick. All the mandated diseases reported were evaluated.

For the cohort portion, all persons in the state of Ohio, starting from January 1, 2010, to December 5, 2014, were included. The rationale for selecting this time frame included that the influenza genome seemed relatively stable as indicated by no change in major components of the influenza vaccine during that time (http://www.cdc.gov/flu/about/season/vaccine-selection.htm/). Also rates of international air travel were fairly consistent, and the disease reporting system as an informatics process was fully developed. Another rationale for selecting the time period selected to be studied was that enough time had passed to have most of the histories and lab tests reported into the system. Cases with incomplete or missing critical variables were excluded (Figure 2). Meningococcal diseases, mumps, measles, and dengue were not included in the final analysis because of low occurrence and inadequate travel history. The nondiseased proportion of the Ohio population at the midpoint of the study was the controls. Only confirmed reports were considered cases for the study.

Prospective field methods typically consisted of routine public health steps taken to report and control communicable disease. These steps included completing the CDC case surveillance report forms for malaria or viral hepatitis or the Ohio Department of Health's IH case report form. All of these forms include a travel history. Cases were classified as confirmed or suspected. For IH only two classes exist: confirmed or not a case. For a hospitalized person with clinical symptoms consistent with influenza to be classified as confirmed rather than suspect, they had to be laboratory confirmed or be epidemiology linked to a confirmed case. HA classification includes a suspect classification and confirmed or not a case. Malaria has two case classifications of suspected (detection of* Plasmodium* species by rapid diagnostic testing) or confirmed by microscopy. Field methods also included contacting the state public health agency immediately if a case of malaria had no recent history of overseas travel as this may mean that local transmission from infected person to vector to person has occurred. For influenza, public health management includes reminders to physicians not to use aspirin to treatment infants, children, or teenagers because of the risk of Reye syndrome. The use of antivirals can be started within the first two days of illness to reduce the severity and shorten the duration but this depends on the susceptibility of the virus to specific antiviral medications. Isolation is usually impractical for influenza. For HA, public health management includes exclusion of cases from food service or child care occupations for 10 days after initial onset of symptoms. If there is an outbreak of HA postexposure prophylaxis of immune globulin or HA vaccine should be considered for all previously unvaccinated close personal contacts, common source exposures.

### 2.3. Nested Case Control

**Figure 1:**
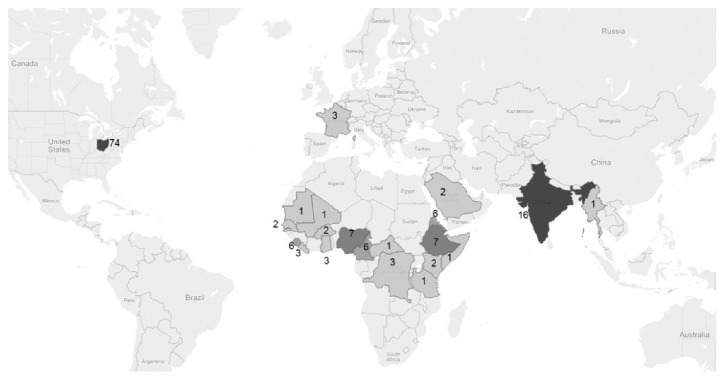
Country visited by world travelers before being confirmed with malaria in Ohio from 2010 through 2014.

For the nested case control portion those with the selected disease were compared to those who did not have the disease. Diseases that were considered for evaluation included dengue, IH, HA, malaria, measles, meningococcal diseases, and mumps. These diseases were selected from the entire set of communicable diseases mandated to be reported to public health because we hypothesized them to impact air travel. The data was analyzed using IBM SPSS Statistics Version 22 Release 22.0.0.0 64-bit edition. Maps were created using Tableau Public Version 8.3.3 (Figure 1). Travel histories are required as part of the disease reporting system for many of those diseases but were mostly complete for cases of HA, IH, and malaria. ODRS data of notifiable diseases were downloaded in an unidentified format with respect to recent history of air travel (conveyance). For the time period of this study, the system typically reported out only one country visited per disease occurrence. In this research study on infectious diseases in Ohio, HA was considered autochthonous (endemic), IH were considered seasonal, and malaria was considered nonendemic.

It was assumed that international travelers would travel at least once and relatively close in time after the issuance of the passport and only enumerated the passports for the study period (ignoring they are good for 10 years). While this may lead to slightly underestimating the number of exposed controls the assumption that passports issued correlate with international travel is based upon the presumption that the costs related to passports rule out most of those who obtain passports for nontravel reasons and that any error would be minimized by the long time span of the study. Further, US passports for persons aged 16 and older are valid for 10 years. We considered US passports issuance as if they were issued without replacement, counting all the passports issued during the study period as representative for the entire population at the study midpoint. The number of enplanements based on boarding to estimate the number of domestic travelers is subject to several biases. Some air travelers enplane even on the same flight more than once and may change conveyance types more than once on the same trip. Domestic travel based on enplanements including international travelers and postarrival from international ports of entry to their final destination would increase the number of enplanements for those passengers. An assumption that international travel was mostly by air would decrease the number of travelers from endemic areas as some come by sea. Histories only provided one country visited; although this information may have appeared in the notes section it was not feasible to extract the notes in the time allotted for this study. Only confirmed cases of malaria, HA, and IH were included. For the diseases selected, the surveillance system had adequate records of histories taken. Case histories were taken in a prospective manner with cases directly observed and exposures measured directly over the 60 months of study. The remaining population exposure metrics allowed for measurement of controls' exposure.

### 2.4. Data Analysis

Descriptive statistics including frequencies, recoding nominal variables, odds ratio, risk ratio with attributable risk, ratio of relative risk of endemic and nonendemic disease, and logistic regression models were developed [29]. For the controls, international travel was determined by the number of passports issued in Ohio from 2010 to 2014 by the US Department of State, Bureau of Consular Affairs, US Passports & International Travel. The number of controls in the population at large was corrected for by the number of cases with reported international or domestic travel (for HA domestic travel was obtained by history). The number of enplaned passengers in Ohio for all airports was retrieved from publicly available Federal Aviation Administration (FAA) data for passenger boarding from the Air Carrier Activity Information System (ACAIS) from calendar year 2012. This year was chosen as it was the temporal study midyear. The Greater Cincinnati Airport which is in Northern Kentucky was also included. The rate of enplanement was assigned as high if there were more than 40,000 passengers per year. The assignment of the rate of annual enplanement was done using the largest natural breakpoint in histogram of total airport enplanements. The airports with the enplanements higher than 40,000 annual included Cleveland-Hopkins International, Port Columbus International, Cincinnati/Northern Kentucky International, James M. Cox Dayton International, Akron-Canton Regional, Toledo Express, and Youngstown-Warren Regional. Since Ohio counties are of similar size, high density metropolitan areas were designated as counties in Ohio with a population of over 300,000 including Butler, Cuyahoga, Franklin, Hamilton, Lorain, Lucas, Montgomery, Stark, and Summit. Counties with the largest number of enplanements included Butler, Clermont, Cuyahoga, Delaware, Franklin, Greene, Hamilton, Lorain, Lucas, Mahoning, Montgomery, Stark, and Summit. The county of residence was used to match the controls to exposure while for cases the county of residence was obtained by history. The population used for Ohio was the midpoint of the study as retrieved from the US Census. Air Travel was defined by the number of passengers enplaned by airport and by proximity of total population to airport. The rationale for using enplanement and an indicator for international air travel (the number of US passports issued) was to differentiate between international and domestic flights and the effect on endemic, autochthonous, and nonendemic disease.

## 3. Results

**Figure 2:**
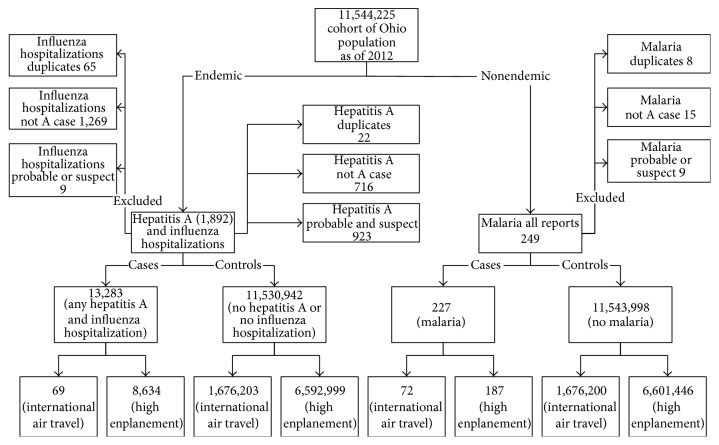
Assignment of cases and controls to exposure to international air travel (derived from total number of US passports issued) and high enplanement (amount of boarding annually).

Ohio females (52.9%) and White race (62.5%) were represented higher than males and non-White race. Hispanic ethnicity was recorded only for 53% of the cases with 51% total being non-Hispanic. We looked at 19,056 cases of dengue, HA, IH, malaria, meningococcal bacteria, and mumps. Figure 1 shows the confirmed cases of malaria all with nonautochthonous travel with the most having traveled to country being India followed by Nigeria, Ethiopia, Cameroon, Eritrea, and Sierra Leone. Figure 2 shows the assignment of cases and controls to exposure to international air travel (derived from total number of US passports issued) and high enplanement (amount of boarding annually).  Table 1 shows the risk of enplanement and international travels for malaria, HA, and IH. International travel's effect on nonendemic disease as represented by malaria (2.7) versus endemic disease as represented by IH and HA (0.031) provided a ratio of relative risk of 87.1 (95% CI 55.8–136). For persons in proximity of an airport with high enplanement the ratio of relative risk of endemic HA and influenza versus nonendemic malaria was protective 0.397 (0.282–0.559). Modeling of malaria, HA, and IH regressed on the following independent variables: female sex, Hispanic ethnicity, non-White race, large metropolitan area, and any history of travel (Table 2). Malaria cases in Ohio from 2010 through the 2014 year showed no statistical change using trend analysis: 51, 46, 53, 59, and 38. However, despite no overt increase in the public health burden of malaria cases, the number of malaria cases was analyzed in relation to US passports issued from 2010 through 2014 showing a 0.69 increase for every one thousand passports issued to Ohio residents in Ohio (*y* = 22.27 + 0.69*∗x*).

## 4. Discussion

The majority of Ohio population is White, female, and non-Hispanic ethnicity and it is the same for our study group. The cohort appears representative to the United States. The public health disease reporting system in Ohio is robust and represents the reality of the situation supporting generalizability to areas of the US with “local public health home rule.” This supports a measure of validity to the rest of the world in regard to the risk of air travel and increased risk of infectious disease to nonendemic areas.

Mumps and meningococcal bacteria were excluded because they do not have a travel history in the data set in the surveillance system. For vector-borne diseases we included malaria because the data set had enough information about travel history. For dengue there was some information missing and for that reason we excluded dengue from the analysis. Of the remaining 19,056 cases we considered IH with 77.7% and HA with 9.9%.

Calculation of the risk of disease given exposure to air travel supports targeted efforts to reduce the risks of disease in endemic areas of the world. There are increasing concerns over changing climatic conditions and the spread of vectors. In Ohio as of November 2, 2015, there were 9 cases of chikungunya, 8 cases of dengue, and 32 cases of malaria (Ohio Department of Health Ohio Arbovirus Surveillance Update, November 2, 2015). While none of these cases were autochthonous (or indigenous) this study supports remediation of vector propagation in relation to changing global climate conditions. The ability of mosquitoes to become established in a new region is directly related to their adaptability to local climes and/or to changing climate conditions. Increased rainfall and rising temperatures could increase breeding sites. Recognition of the issues surrounding travel and transmission can allow international travel to continue. In our experience the residences within our jurisdiction support public health efforts globally such as the United Nations' Mission in Liberia, the public health response to Ebola in West Africa, building of Ebola treatment facilities, and the development of fresh water wells in Sierra Leone. These supports are only possible through international air travel.

The infectivity of selected diseases in ascending order is hepatitis C, Ebola, HIV, SARS, mumps, and then measles [30]. Influenza has an infectivity rating from 1.8 to less than 1 [31]. Our public health jurisdiction, Greene County, is in the middle of 4 statistical metropolitan areas and is bounded by three national interstates (I-70, I-71, and I-75). The county is home of the largest military reservation in Ohio, the Wright Patterson Air Force Base, as well as being home to institutions of advance learning known for their international travel and students. In Ohio, disease related concerns over the past decade have included measles, SARS,* Fusarium*,* Salmonella*, mumps, novel influenza, botulism, campylobacteriosis, multidrug resistant tuberculosis, malaria, and Ebola. The state's strategic plan is focused on emerging infections because of a large outbreak of mumps and measles in Ohio during the year 2014.

One strength of this study is the assumption of autochthonous HA and seasonal IH. When we compared the rate of travel among these diseases, the rate of HA was 10 times greater in persons with a history of international travel. With IH it was even greater at over 50 times. Our assumption of endemic HA and seasonal IH is justified given the low rate of travel among these diseases.

Novel influenza was not included in this study for several reasons. Ohio has a county fair system that allowed for several sporadic “novel” introductions to the region of novel influenza related to exposure to swine during this study's time frame. The Centers for Disease Control and Prevention's (CDC) case count of human infections with H3N2v for Ohio was 0, 0, 107, 1, and 2 from 2010 through 2014, respectively (http://www.cdc.gov/flu/swineflu/h3n2v-case-count.htm/). The display of swine allowed for close human to swine interaction and caused an increase during the year 2012. While later the H3N2 variant swine influenza strain was added as a component to the influenza vaccine another aspect of potential confounding was introduced with the emergence of the H3N2 Switzerland type that caused an increase in hospitalizations from this strain; despite being different the swine influenza strain of H3N2 variant called Texas that had been novel in 2012 and by 2014 was no longer considered novel.

Why would high enplanement increase the risk for malaria? Enplanement is mostly made up of domestic flights. International air travel is represented by passports for the controls in the cohort and in the cases; the travel history is indicative. Given our rationale for using enplanement as an indicator for domestic travel in the controls and a negative history of travel through case interviews, high enplanement captures international air travelers as well as domestic travel. Low enplanement areas represent mostly regional and domestic flights. A likely scenario is that the students, families, and friends the traveler visits in the countries shown in Figure 1 suggest a traveler who has come to an area with high enplanement such as a major metropolitan area for business or education. One could hypothesize a younger adult, a risk taker traveling around the world and perhaps being in areas where mosquitoes could prevail. The longer potential incubation time for malaria of well over a month would suggest that a percentage of persons traveling from endemic areas are going to come down with malaria. Why would international travel be protective for HA and influenza? The vaccine preventable disease of influenza would be a requirement for entry into the United States. The cost of international travel would also suggest that the traveler perhaps is from a higher socioeconomic strata than most persons who come down with the disease and cannot afford the expense associated with international travel and would have less need to have the influenza vaccine.

## 5. Conclusions

International air travel in regard to risk of infectious disease was protective, but when considering endemic versus nonendemic diseases in the state of Ohio it is shown that the risk was higher for nonendemic diseases. The air travel associated risk for malaria was 2.7 (2.07–3.62) while the rate of HA and IH was 0.031 (.02–.04). The risk of influenza, HA, and malaria combined was higher in airports with greater enplanement 1.4 (1.34–1.44). The risk for malaria was significantly higher in airports with greater enplanement 3.5 (2.5–4.9) than the risk for HA and influenza 1.39 (1.34–1.44). There is a risk of malaria of 1,494 (320–6,975), for HA 11.8 (6.15–22.9) and for IH 4.5 (1.05–19.4), with history of air travel. International air travel by infected travelers increases infectious disease to nonendemic areas by a ratio of relative risk of nonendemic malaria versus endemic HA and IH by 87.1 (55.8–136). There is not an increasing trend over time of vector versus nonvector related diseases. Endemic disease is lower than nonendemic disease relative to domestic travel, as shown by the ratio of relative risk of HA and influenza versus malaria 0.397 (0.282–14 0.559). Malaria is correlated with international travel with the number of malaria cases in relation to international showing a .69 increase for every one thousand passports issued to Ohio residents.

## 6. Recommendations

Taking a travel history of persons with communicable infectious diseases with a latent onset longer than the travel time should be a standard for all diseases reported to the public health surveillance systems. All the major destinations the traveler has been to during the incubation period should be included in the history (not just a single country). Passengers with nonendemic diseases should alert health officials once they arrive to their destination. People who want to travel to regions with certain endemic diseases should make sure their vaccinations are up to date and all the necessary prophylaxis and other precautions are complete prior to departure. Coordination with other agencies to ensure tracking of returning passengers should be strengthened through existing coordinating agencies. Specifically, more resources should be provided to CDC's Division of Global Health Protection at this component of the CDC's Center for Global Health works with ministries of health and other partners to protect the health and improve the well-being of people globally by building public health capacity to prevent disease, disability, and death from communicable and noncommunicable diseases. In support of this, screening at airports for Ebola was carried out from August to November 2014 of estimated 80,000 travelers of 12,000 that were en route to the United States, none of which were reported as symptomatic with Ebola during travel since these procedures were implemented [32]. Screening from passengers from hot zones has to be done in a culturally competent manner to ensure risks and benefits of cooperation are fully understood to minimize bias in histories. The focus on medical treatment facilities in conjunction with control of moderators of disease, for example, vectors, should be increased.

Our research shows that air travel increases the risk of infectious disease indicating that banning of flights could work in slowing down the spread of infectious disease. However, we are not convinced of this despite the fact that “school closure is the single most effective Non-Pharmaceutical Intervention” [33], but a strategy to mitigate outbreaks is needed. The alternative is to closely monitor suspected passengers and making sure that all passengers going to hot zones take all the extreme precautions. However, air traveler screening is ineffective for diseases with long incubations and inaccurate reporting by travelers [34]. Screening could be improved by customizing screening to the suspect agent. The current Ebola outbreak has killed 8,626 people as of January 18, 2015. In our personal communications with colleagues in West Africa the statement was made that “Ebola may rise again in Montserrado complacent that Ebola has passed in desire to return to the old ways only to bring death.” The World Health Organization proposes Ebola reform includes a “dedicated contingency fund to support rapid responses to outbreaks [35].”

In regard to malaria, our study confirms and extends a recent review that found risk factors associated with malaria were “non-use or inappropriate use of chemoprophylaxis, age, delay in seeking care, incorrect treatment, delay in diagnosis, infection with* Plasmodium falciparum*, non-immunity, travelling as a tourist, and sex [36].” The case fatality rate ranged from 0.2% to 3%. Other researchers found younger travelers to be higher risk takers during traveling [37], where others found travelers older than 70 years of age at greatest risk of dying [38]. Clearly malaria chemoprophylaxis and counseling on risk avoidance of malaria are indicated for international travelers to endemic areas.

Our recommendation in regard to keeping international air travel safe is to promote the safe use of air travel to mitigate outbreaks of infectious disease as well as to have a thoughtful and careful response to ongoing disease occurrences involving air travel. In the face of emerging infectious disease threats and rising antibiotic resistance we can be comforted by the fact that traditional public health efforts such as isolation and quarantine can work to stop people from becoming infected and can decrease morbidity and mortality if they are carefully and competently implemented. Further research is needed to study the beneficial anecdotal effects of air travel, such as what we have seen from our brave residents traveling to help mitigate Ebola in West Africa.

## Figures and Tables

**Table 1:** Risk ratios of infectious diseases by exposure to high enplanement or international air travel in Ohio from 2010 through 2014 (total population = 11,544,225) with 95% confidence intervals.

Status	Exposure	Exposed cases	Exposed controls	Unexposed cases	Unexposed controls	Risk ratio	Lower	Upper
Any hepatitis A, influenza hospitalization, or malaria	International travel	141	1,676,131	13,369	9,854,584	0.062	0.03	0.07
Malaria	International travel	72	1,676,200	155	9,867,798	2.735	2.07	3.62
Any hepatitis A, influenza hospitalization	International travel	69	1,676,203	13,214	9,854,739	0.031	0.02	0.04
Any hepatitis A, influenza hospitalization, or malaria	High enplanement	8,821	6,592,812	4,689	4,937,903	1.408	1.36	1.46
Malaria	High enplanement	187	6,601,446	40	4,942,552	3.500	2.49	4.92
Any hepatitis A, influenza hospitalization	High enplanement	8634	6,592,999	4,649	4,937,943	1.390	1.34	1.44

*Note*. Hepatitis A and influenza hospitalization in this research were considered endemic or seasonal, respectively, while malaria was considered a nonendemic, nonautochthonous disease. Airports with high enplanement defined as greater than 40,000 (boarding per year) serving Ohio include Cleveland-Hopkins International, Port Columbus International, Cincinnati/Northern Kentucky International, James M. Cox Dayton International, Akron-Canton Regional, Toledo Express, and Youngstown-Warren Regional. International travel was defined as residents of Ohio issued US passports from 2010 through 2014.

**Table 2:** Logistic regression of all confirmed cases of malaria, hepatitis A, and influenza. Hospitalizations in the cohort of the total population of Ohio from 2010 through 2014 (suspect and probable reports were excluded from the cases).

Model	Exposure	*B*	Exp(*B*)	95% CI for Exp(*B*)		Model
Infectious disease				Lower	Upper	*R* square
Hepatitis A *774 controls* *162 cases*	Female	−0.226	0.798	0.56	1.14	0.121
	Hispanic	−0.357	0.700	0.29	1.71	
	Non-White	−0.613	0.542	0.32	0.91	
	History of travel	2.475	11.877	6.15	22.94	
	Metropolitan area (>300,000)	0.566	1.761	1.21	2.57	
	Constant	−1.760	0.172			

Influenza hospitalizations *3,171 controls* *6,791 cases*	Female	0.065	1.068	0.98	1.16	0.002
	Hispanic	−0.230	0.795	0.64	0.99	
	Non-White	0.076	1.079	0.97	1.19	
	History of travel	1.507	4.511	1.05	19.39	
	Metropolitan area (>300,000)	−0.101	0.904	0.83	0.99	
	Constant	0.766	2.150			

Malaria *9,783 controls* *179 cases*	Female	−0.741	0.476	0.33	0.69	0.364
	Hispanic	−0.801	0.449	0.17	1.19	
	Non-White	1.880	6.554	4.26	10.09	
	History of travel	7.310	1494.4	320.17	6975.48	
	Metropolitan area (>300,000)	0.378	1.459	0.93	2.29	
	Constant	−5.218	0.005			

*Note*. Exposures were assessed retrospectively from case records and matched to controls by county of residence.

## Additional Points

Air travel and enplanement separately increased the risk of influenza hospitalizations, hepatitis A, and malaria, with malaria increase by 69% for every one thousand passports issued.
